# Testing if Micro-CT Is Capable of Quantitating the Extent of Proteoglycan-Aggrecan Induced Axial Spondyloarthritis in Mice

**DOI:** 10.3389/fimmu.2021.681217

**Published:** 2021-07-05

**Authors:** Qing Han, ZhaoHui Zheng, Qiang Liang, Kui Zhang, FengFan Yang, XiangHui Fu, Xing Luo, Jin Ding, Ronghua Xie, WenXiao Zhu, Ping Zhu

**Affiliations:** ^1^ Department of Clinical Immunology, PLA Specialized Research Institute of Rheumatology & Immunology, Xijing Hospital, Fourth Military Medical University, Xi’an, China; ^2^ National Translational Science Center for Molecular Medicine, Xi’an, China

**Keywords:** spondyloarthritis, proteoglycan, aggrecan, BALB/c, animal models

## Abstract

**Objective:**

Injections of proteoglycan aggrecan (PGA) have been reported to induce axial spondyloarthritis (ax-SpA) in BALB/c mice. It is considered to be a model for radiographic ax-SpA. However, evaluation of the extent of axial disease by histopathological assessment of every intervertebral space is labor-intensive. The objective of our paper is to test the feasibility of Micro Computed Tomography (Micro-CT) in rapidly enumerating the number of intervertebral spaces affected in each mouse.

**Methods:**

Arthritis was induced in BALB/c mice by intraperitoneal injections of PGA. Involvement of several spinal segments, and selected sacroiliac and hip joints were evaluated by histopathology. The involvement of all intervertebral spaces, sacroiliac and hip joints was evaluated by Micro-CT.

**Results:**

BALB/c mice injected with PGA developed histopathology of SpA-like axial lesions, including spondylitis, sacroiliac joint arthritis and hip joint arthritis. Micro-CT allowed us to clearly enumerate the number of lesions in each mouse.

**Conclusion:**

Micro-CT allows quantitative assessment of the extent of axial involvement in PGA-induced mouse spondylitis. This can be a useful tool in assessing therapeutic interventions.

## Introduction

Axial Spondyloarthritis (ax-SpA) is a chronic inflammatory disease that affects the spine and sacroiliac joints (SIJs). The prototype is Ankylosing Spondylitis (AS) (1). Although the exact pathogenesis of SpA is not known, in AS, Human Leukocyte Antigen (HLA-B27) is a major genetic contributor (2). Tumor necrosis factor (TNF) is one of the main disease-causing cytokines (3, 4). The other known cytokine axis is the interleukin (IL) -23/IL-17 axis (5, 6). Proteoglycan (PG) is a normal component of human and mouse cartilage tissue. Immunizing BALB/c mice with human PG in presence of adjuvants turns it into an autoantigen (7). About 70% of PG-immunised BALB/c mice develop SpA (PG-induced spondylitis, PGISp), with a disease pathology and progression very similar to human AS (8). The success of inhibitors of TNF, IL-17 and Janus Kinase (JAK) in human patients have led to an explosion of clinical therapeutic trials of other modalities. The PGISp mouse model is potentially a useful screening tool for potential novel therapeutic agents. However, being in the spine, assessment of individual spinal segment by histopathology will be labor-intensive. The purpose of this study was to test the feasibility of Micro-CT as a rapid and accurate tool in quantitative evaluation of the extent of involvement of spine, SIJs and hip joints in this mouse model. The first step of our experiments was to validate the model in our laboratory using histopathology and cytokine assays. The final step was to test if Micro-CT could distinguish each affected intervertebral disc (IVD) and joints from healthy controls (HC).

## Methods

### Experimental Animals

Specific pathogen-free (SPF) BALB/c mice purchased from the Experimental Center of the Fourth Military Medical University were placed in a pathogen-free facility. All animal experiments are conducted in accordance with the National Institutes of Health (NIH) guidelines and the guidelines and regulations of the Experimental Animal Research Center at the Fourth Military Medical University.

### PGA-Induced SpA Mouse Model

BALB/c female mice (6∼8 weeks) were used as experimental animals. Model group (n = 15) was injected with 100g PGA (dissolved in 100uL complete Freund’s adjuvant) at time zero. The same dose of PGA (dissolved in 100uL incomplete Freund’s adjuvant) was injected intraperitoneally at week 3 and 6 (9). The control group (n = 15) was given intraperitoneal injection of Phosphate Buffer Saline (PBS) and the same doses of adjuvants at the same time schedule as the model group. Mice were sacrificed at week 36 after the third injection. For the care and use of laboratory animals, all procedures used in this study followed the National Institutes of Health guidelines. The institutional Animal Care Committee approved the animal testing program. The Animal Experiment Administration Committee of the Fourth Military Medical University approved all protocols.

### Histopathological Examination

The mice were anesthetized and euthanized 36 weeks after the first PGA injection. Tissue samples were fixed in 10% formalin, decalcified in EDTA, and embedded in paraffin. The sections were dewaxed with xylene, dehydrated in a series of graded alcoholic solutions, and then stained with hematoxylin and eosin (H&E).

### Micro-CT Imaging

Mice were anesthetized and Micro-CT of the entire skeletons obtained by a Micro-CT machine using a protocol provided by the manufacturer (Inveon; Siemens Healthcare Solutions, Knoxville, Tenn, USA). A companion software reconstructed the images into a 40 m voxel 3D format (Inveon Acquisition Workplace; IAW version 1.5, Siemens Medical Solutions). The effective pixel size (EPS) of the scanning image was 27.8~55.6μm, the scanning time was 30 minute~1 hour, and the average number of scanned slices was 1537.

### Cytokine Analysis of Mouse Serum Samples

Blood samples were obtained from isoflurane-anesthetized animals by heart puncture 36 weeks after the third injection. Serum samples were stored at -70°C for batch analysis. A Luminex magnetic beads multiple assay system (R&D Systems, Minneapolis, MN, USA) was used to assay the samples for TNF-α, IL-6, IL-17A, and IL-10, according to manufacturer’s instructions.

### Statistical Analysis

The results were expressed as the mean ± standard (SD) of the clinical arthritis score. Normally distributed group comparisons are performed using the T-test, non-normally distributed groups using the Man-Whitney U test for. Variance bidirectional analysis (ANOVA) was used to analyze the influence of PGA on clinical scores over time, and then Tukey post-mortem test was performed. All analyses were performed using version 19.0 of SPSS software (SPSS Inc., Chicago, Illinois, USA) and version 7 of GraphPad Prism software (GraphPad Software, San Diego, California, USA).

## Results

We did not observe any joint swelling in the peripheral joints. The following results were confined to the spine, the sacroiliac and the hip joints.

### Histopathological Features of BALB/c Mice Induced by PGA

**Figure 1:**
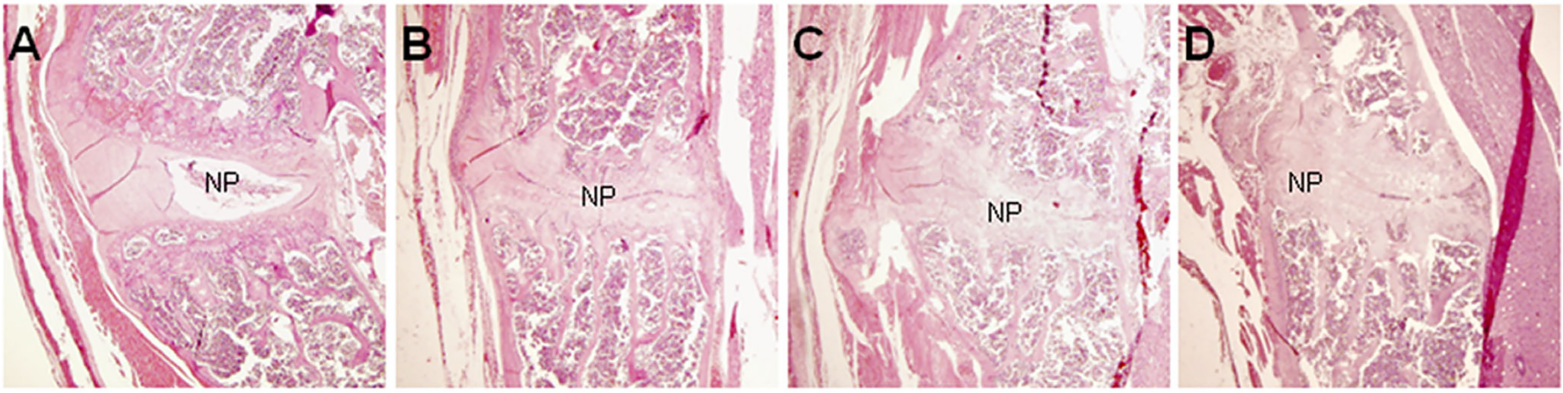
Pathological changes of spondylitis in BALB/c mice induced by PGA injections (HE, × 40). At the center of each figure is the IVD. **(A)** HC mouse; **(B)** Extensive narrowing of intervertebral space in a PGISp mouse; **(C)** More extensive narrowing of intervertebral space; **(D)** Bridging bone formation in part of the intervertebral space; NP= nucleus pulposus.

**Figure 2:**
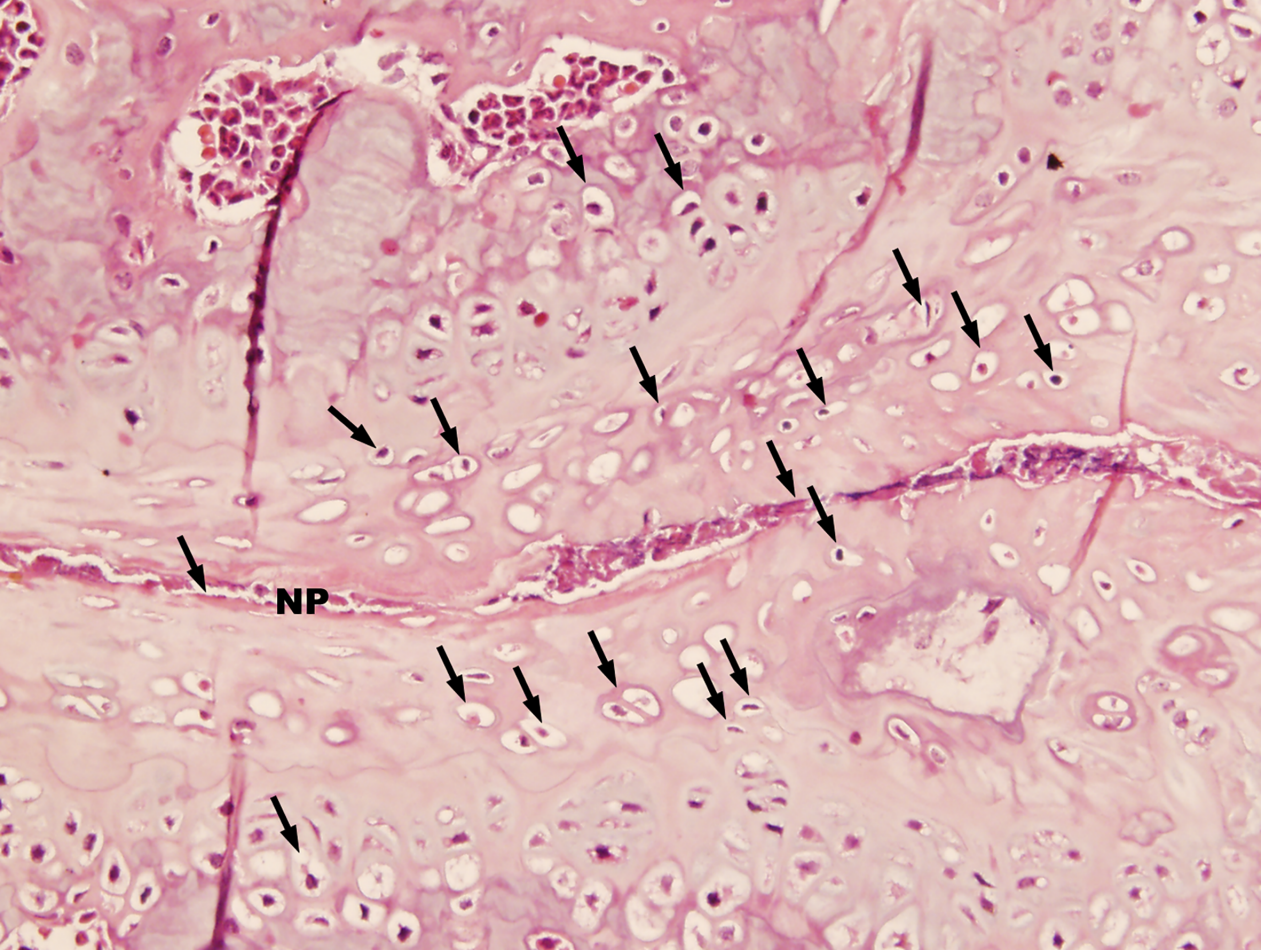
Advanced pathological changes of an intervertebral space in a PGISp mouse (HE, × 400). A large number of chondrocytes (black arrows) appeared next to the very much narrowed intervertebral space. Part of the intervertebral space seemed to have disappeared.

Histopathological analysis was performed on mice 36 weeks after the first injection of PGA (n = 10). Control mice were shown in **Figure 1A**. In control mice, the nucleus pulposus was wide and the adjoining bone surfaces were smooth. In PGISp mice, we observed extensive narrowing of the intervertebral space **(** **Figures 1B, C** **)**, and in certain areas the intervertebral space was bridged by bone **(** **Figure 1D** **)**. When observed at ten times higher magnification, many chondrocytes were observed next to the much narrowed intervertebral space **(** **Figure 2** **)**.

### Micro-CT Results

**Figure 3:**
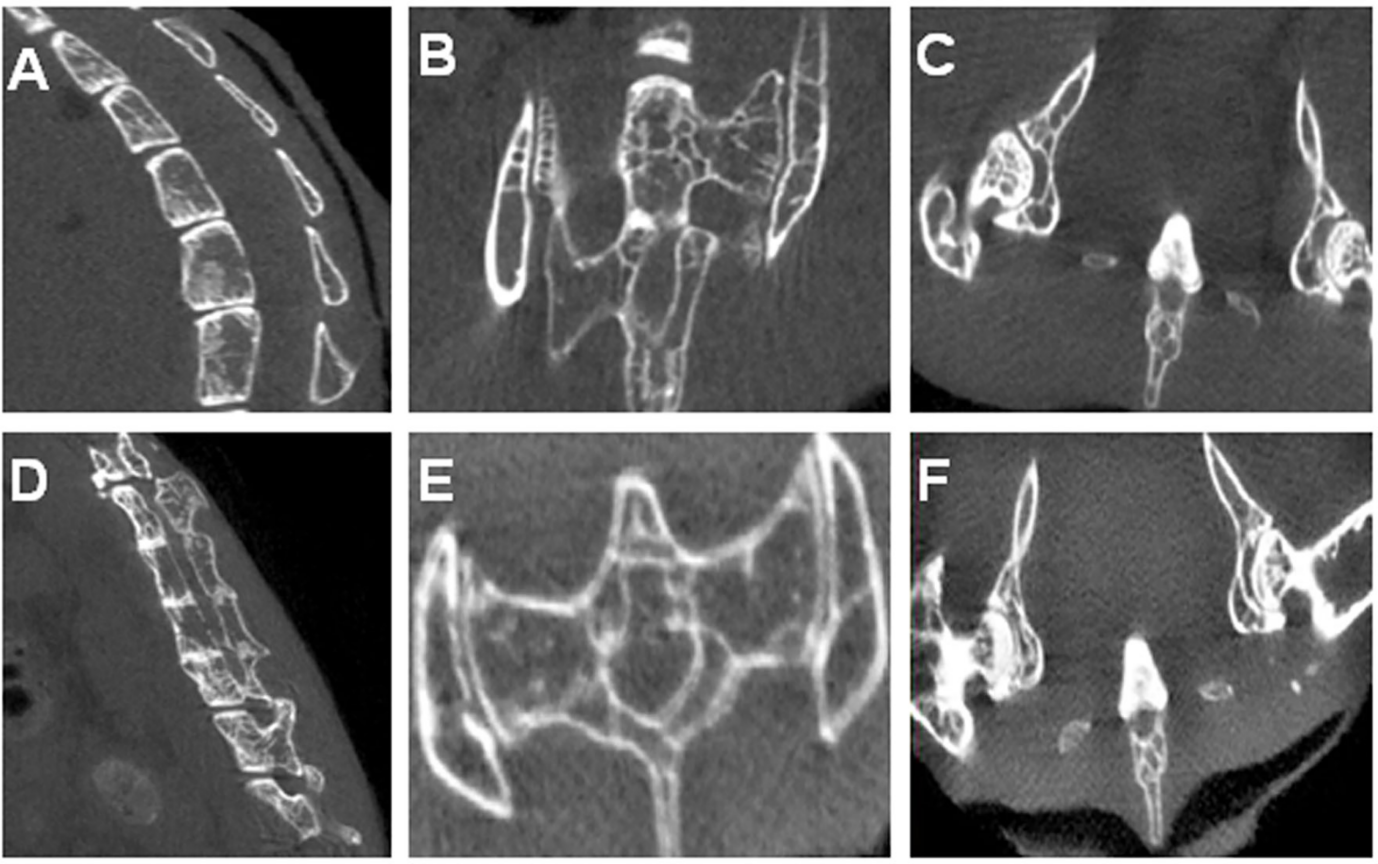
Micro-CT results: **(A)** A control mouse showing physiological spinal curvature, intact bone, smooth articular surface, and substantial intervertebral space; **(B)** SIJs of a control mouse; **(C)** Hip joints of a control mouse; **(D)** Spine of an PGA-induced model mouse showing loss in curvature, irregular and sclerotic articular surfaces, and narrowing of intervertebral discs. Some of the vertebral bodies appeared fused; **(E)** SIJs of an PGA-induced model mouse showing irregular joint surfaces; **(F)** Hip joints of an PGA-induced model mouse showing irregular joint surfaces and narrowing of joint spaces.

**Figure 3A** shows several control vertebral bodies with clearly defined intervertebral space. **Figures 3B, C** show the control SIJs and a hip joint respectively. The joint surfaces appeared smooth, and the joint space distinct. In PGISp mice group, the intervertebral space became much narrower **(** **Figure 3D** **)**. The surfaces of the SIJs appeared irregular **(** **Figure 3E** **)**. The space of the hip joints also became much narrower **(** **Figure 3F** **)**.

### Serum Cytokine Analysis

**Figure 4:**
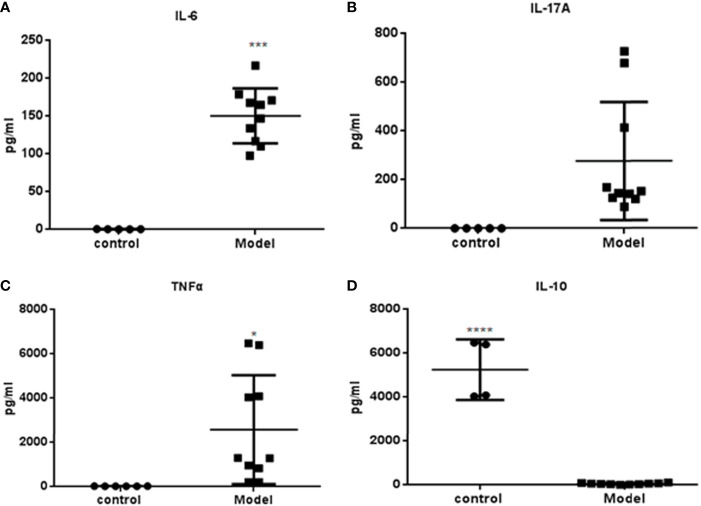
Cytokines measured by Luminex determination (R&D Systems, USA): Serum was obtained from BALB/c mice immunized with PGA or PBS at week 36. **(A)** IL-6; **(B)** IL-17A; **(C)** TNF-α; **(D)** IL-10. The expressions of IL-6, IL-17A and TNFα were higher in the PGA-induced model group compared with those in the control group. The expression of IL-10 in the PGA-induced model group was lower than that in the control group (control group = 4, PGA-induced model group = 9). *p < 0.05; ***p < 0.01; ****p < 0.001, compared with control group.

Serum samples (n=9) were obtained at week 36 and cytokine levels were measured. The expressions of IL-6, IL-17A and TNF-α were significantly higher in the PGA-induced mice compared with those in the control group **(** **Figures 4A–C** **)**. Compared with the control group, the expression of IL-10 in mice that induced by PGA was significantly decreased **(** **Figure 4D** **)**.

### Evaluation of Number of Affected Intervertebral Space and Affected SIJs and Hip Joints as Visualized by Micro-CT

All the intervertebral spaces, the sacroiliac and hip joints appeared normal in the control mice. In **Table 1**, we designate the observation of each intervertebral space of each PGA-induced model mouse as positive or negative. A positive sign indicates an abnormal appearing space/joint. A negative sign indicates a normal space/sign. Data of sacroiliac and hip joints were not available in 3 mice because we only had X-ray images of those joints in those mice. The resolution in X-ray images were too low to be interpreted.

**Table 1:** X-ray/Micro-CT assessment of individual intervertebral space as well as sacroiliac and hip joints in PGISp mice.

X-ray	IVD Inflammation	M1	M2	M 3	M 4	M 5	M 6	M 7	M8	M 9	M10	M 11	M12	M 13	M 14	Numbers of Positive mice
	C1-C2	–	–	–	–	–	–	–	–	+	+	+	+	+	+	6
	C3-C4	–	–	–	–	–	–	–	–	+	+	+	+	+	+	6
	C4-C5	–	–	–	–	–	–	–	–	+	+	+	+	+	+	6
	C5-C6	+	+	–	–	–	–	–	–	–	–	–	+	+	+	5
	C6-C7	+	+	–	+	–	–	–	–	–	–	–	+	+	+	6
	T1-T2	+	+	–	–	–	–	–	–	–	–	–	–	–	–	2
	T2-T3	+	+	+	+	+	+	+	–	–	–	–	–	–	–	7
	T3-T4	+	+	+	+	+	+	+	–	–	–	+	–	–	–	8
	T4-T5	+	+	–	–	–	–	–	–	–	–	–	+	+	–	4
	T5-T6	+	+	–	–	–	–	–	–	–	–	–	+	+	–	4
	T6-T7	+	+	+	+	–	+	+	–	+	+	+	+	+	+	12
	T7-T8	+	+	+	+	–	+	+	–	+	+	+	+	+	+	12
	T8-T9	+	+	+	+	–	+	+	–	+	+	+	+	+	+	12
	T9-T10	+	+	+	+	–	+	+	–	+	+	+	+	+	+	12
	T10-T11	+	+	+	+	–	+	+	–	+	+	+	+	+	+	12
	T11-T12	+	+	+	–	+	–	+	–	+	–	+	–	+	–	8
	L1-L2	+	+	+	+	+	+	+	+	+	+	+	+	+	+	14
	L2-L3	+	+	+	–	+	–	+	+	+	–	+	–	+	–	9
	L3-L4	+	+	+	+	+	+	+	+	+	+	+	+	+	+	14
	S1-S2	+	+	+	+	+	+	+	+	+	+	+	+	+	+	14
	S2-S3	+	+	+	+	+	+	+	+	+	+	+	–	–	–	11
	S3-S4	+	+	+	+	–	+	+	+	+	+	+	+	–	–	12
**Micro-CT**	R-SIJ	+	+	+	–	NA	NA	NA	+	+	+	+	+	–	–	7
	L-SIJ	+	+	+	+	NA	NA	NA	+	+	+	+	+	–	–	8
	R-hip	+	+	+	+	NA	NA	NA	+	+	+	–	–	–	–	7
	L-hip	+	+	–	–	NA	NA	NA	–	+	+	–	–	–	–	4
	Shoulders	–	–	–	–	NA	NA	NA	–	–	–	–	–	–	–	0
	Ankles	–	–	–	–	NA	NA	NA	–	–	–	–	–	–	–	0

M, PGISp mouse; IVD, intervertebral disc; C, cervical spine; T, thoracic spine; L, lumbar spine; S, sacral vertebrae; R-SIJ, right sacroiliac joint; L-SIJ, left sacroiliac joint; R-hip, right-hip joint; L-hip, left-hip joint; NA, data not available.

All PGISp mice showed changes in some intervertebral spaces. Pathology mainly concentrated in T6∼T12, L1∼L2, L3∼L4, S1∼S2. Changes in SIJs and hip joints were observed in 100% and 57% of PGA-induced model mice **(** **Table 1** **)**.

## Discussion

The IVD is a complex fibrochondrocyte structure containing chondrocytes and fibroblast-like cells embed in a relatively vascular environment. The extracellular matrix of IVD is mainly composed of collagen and PG, the nucleus pulposus of type II collagen and aggregative PG, and the anulus fibrosus of type I collagen and aggregative PG. PG is also present in articular joints. In theory, an autoimmune response towards PG will lead to inflammatory diseases in the intervertebral discs as well the peripheral joints. Indeed, PG induced arthritis is a well-established mouse model (10). It is different from collagen-induced mouse arthritis. In collagen-induced arthritis, the highest incidence is in DBA/1 mice. In PG induced arthritis, the highest incidence is in the BALB/c mice. The incidence of arthritis in the progeny of BALB/c mice hybridization with DBA/2 mice was only 43.5%, suggesting that the arthritis was related to the Major Histocompatibility Complex (MHC) gene, although the specific susceptibility gene loci of BALB/c mice have not yet been determined (11, 12).

In all reports of PG induced arthritis, both peripheral and axial arthritis are present. However, even in BALB/c mice the phenotype of the arthritis varies among subclones of mice in the same institute (13). The particular colony we immunized did not show any peripheral arthritis, but showed disease in the intervertebral disc, the hip and the SIJs. Arthritis mice also showed higher levels of TNF-α and IL-17A in the sera. These features are similar to patients with AS. Although the arthritis in AS is not caused by an autoimmune response against PG, there is probably some similarity in the downstream processes. Hence, the mouse model can be used for screening of potential therapeutic agents. What is needed is an accurate and convenient tool to quantitate the extent of disease. Being in the spine, disease involvement is much more difficult to observe compared to the peripheral joints. With at least twenty IVDs, it will be labor-intensive to assess each by histopathology. Our results with Micro-CT demonstrate that it provides clear images of the IVD, the sacroiliac and the hip joints.

A major limitation of this paper is that it does not address the pathogenesis of PGA arthritis. The serum cytokine tests and the immunohistology were carried out to ensure it was an inflammatory disease with tissue destruction and changes in cytokines parallel to those with human SpA. The only conclusion which we can be confident are observations from the micro-CT.

## Conclusion

Micro-CT is an accurate and convenient tool to quantitate disease involvement in PGA-induced mouse arthritis in the spine, sacroiliac and hip joints. 

## Data Availability Statement

The raw data supporting the conclusions of this article will be made available by the authors, without undue reservation.

## Ethics Statement

The animal study was reviewed and approved by Fourth Military Medical University.

## Author Contributions

QH, ZZ, QL, and PZ contributed to the conception of the work, completed the first draft, and final version of the manuscript. QH, ZZ, QL, KZ, and PZ contributed to the design of the work. QH, ZZ, QL, KZ, FY, XF, XL, JD, WZ, and RX contributed to the data acquisition and analysis. QH, ZZ, QL, and PZ contributed to interpretation of data. All authors were involved in the manuscript revision and agreed with final approval of the version, and ensured the accuracy of investigation. All authors contributed to the article and approved the submitted version.

## Funding

This study was funded by the National Basic Research Program of China (2015CB553704) and the National Nature Science Foundation Key Research Project of China (2017YFC0909002).

## Conflict of Interest

The authors declare that the research was conducted in the absence of any commercial or financial relationships that could be construed as a potential conflict of interest.
